# Recurrent Leiomyosarcoma With Malignant Transformation

**DOI:** 10.7759/cureus.12672

**Published:** 2021-01-13

**Authors:** Sachini Malaviarachchi, Jayantha Balawardana, Asela Senanayake, Ranga Perera, Sandini Liyanage

**Affiliations:** 1 Clinical Sciences, Sir John Kotelawala Defence University, Colombo, LKA; 2 Surgery, Sir John Kotelawala Defence University, Colombo, LKA; 3 Oncology, Sir John Kotelawala Defence University Hospital, Colombo, LKA

**Keywords:** congenital infantile fibrosarcoma, leiomyosarcom, recurrence, chemotherapy, radiotherapy, responce, assessment results, ultrasound scan, histology, childhood

## Abstract

The soft tissues are comprised of various structures and supportive tissues in the body, including muscle, connective tissues, endothelium, synovium, fat, lymphatics, and fascia. Soft tissue sarcomas may arise in any part of the body. The most common sites are the trunk and the extremities. Fibrosarcoma is the most common non-rhabdomyosarcoma soft tissue sarcoma (NRSTS) in children, in whom two peaks in incidence are observed. The first is in children younger than five years, and the second is in children and adolescents aged 10-15 years. Infantile fibrosarcoma (IFS) is almost exclusively observed in children younger than two years. Many of these sarcomas are congenital. This tumor is locally aggressive, but rarely metastatic, and occurs in the extremity in 70% of patients. A 29-year-old lady presented to the Oncology unit with the fifth recurrence of fibrosarcoma which was transformed into very vascular and high-grade leiomyosarcoma from the fourth recurrence onwards. Initially, it was diagnosed when she was two days old and radiologically diagnosed as lymphangioma. It was documented as a large lump at the right lumbar region with uniform echogenicity and was excised and the postoperative period was uneventful. At the age of 12 years, she presented with a large mass at the right lumbar region over the surgical scar and complete excision done with R0 resection. Histology revealed well-differentiated fibrosarcoma with varying sizes of fibroblasts and no alignment features. The third relapse was at the age of 27. Complete excision was done and histology reported as spinel cell tumor with the possibility of fibrosarcoma or leiomyosarcoma. It was R0 resection and adjuvant treatment was not offered. Tumour recurred over the same scar within a year. Since the tumor is very big with 10 cm craniocaudal and 9 cm wide neoadjuvant chemotherapy is given: IV doxorubicin and IV cisplatin. Surgery was performed after four cycles and found necrotic tissue only. There were no tumor tissues or any form of spindle cells. Due to the aggressive nature, there was no exact solid tumor to assess margins for recommended adjuvant radiotherapy; IMRT. Due to the delay in finding a spacer to keep the bowel away from the radiotherapy field, radiotherapy was delayed and the tumor recurred within six months. It was a radiologically very vascular tumor and she had severe neuropathy and autotoxicity and could not offer any more chemotherapy. The tumor was rapidly growing and was bigger than the last time. Tumor growth was controlled with antiangiogenesis inhibitor; IV bevacizumab and high dose steroids and opioids for pain. After four months, we could not continue bevacizumab due to very high blood pressure and the patient died while waiting for palliative surgery. Childhood fibrosarcoma, including IFS, has classically been treated with surgery alone or with neoadjuvant chemotherapy and surgery. In cases that are not amenable, surgical resection is done upfront. Patients with IFS have an excellent prognosis, with survival rates of more than 90% in some series. A multidisciplinary approach is essential in managing infantile fibrosarcoma as it has a high potency of recurrence during teenage or later in life with malignant transformation. This could have been prevented when the clinicians are well aware of this risk of recurrence and primary surgery has to be planned very carefully with multidisciplinary involvement.

## Introduction

Infantile fibrosarcoma (IFS) is usually found in infants and young children. Sometimes it is also found ultrasonically before birth. Even though it is a rare tumor recurrence is not uncommon. Histopathologically it resembles fibroblasts and they are malignant. The histologic diagnosis is sometimes difficult due to the nature of surrounding soft tissues.

Interestingly they carry a good prognosis. Eighty percent of IFSs are curable and the treatment of choice is surgery. The drawback of surgery is that they are mutilating since the tumor is very big at presentation. According to Daniel Orbach et al. [1], the 10-year survival rate is around 89% with complete excision and neoadjuvant chemotherapy. Treatment decisions would always individualize and a standard treatment plan is made according to initial tumor size and feasibility of achieving adequate resection margins with R0 resection. After resection no adjuvant chemotherapy but active surveillance for group I to II tumors. [1]. According to the Europian Soft Tissue Sarcoma Study Group [2] neoadjuvant anthracycline free and alkylating agents free chemotherapy should be administered to group II patients because they can cause nephrotoxicity and cardiotoxicity in early childhood. Anthracyclines and alkylating agents are kept reserved for use in resistance cases [3].

## Case presentation

A lady presented to the Oncology clinic with a history of severe unbearable pain in the right hypochondriac area. She had a large scar at her right loin area and a history of surgical resection of a mass under the scar when 12 years. Her past medical history revealed that she had a large mass in the same area; tumor resection surgery was done when she was two days old. There was a large solid mass; in the right lumbar region with no direct connection to the right kidney. The right kidney appears to be normal, with uniform echogenicity. No other intra-abdominal masses and tumors were detected, and the tumor excised completely. Histologically it was diagnosed to have IFS. She was disease-free for 12 years following surgery; no adjuvant treatment was given. At the age of 12 years, the tumor has recurred with a large mass. Her records carry fibrosarcoma with spindle cell hyperplasia. Adjuvant therapy was not offered due to complete resection margins.

Pathologically reported that the possibility of either a reaction to chronic inflammation or a well-differentiated fibrosarcoma due to the tissue sample being histologically very cellular, with varying sizes of fibroblasts with no increasing mitosis.

**Figure 1:**
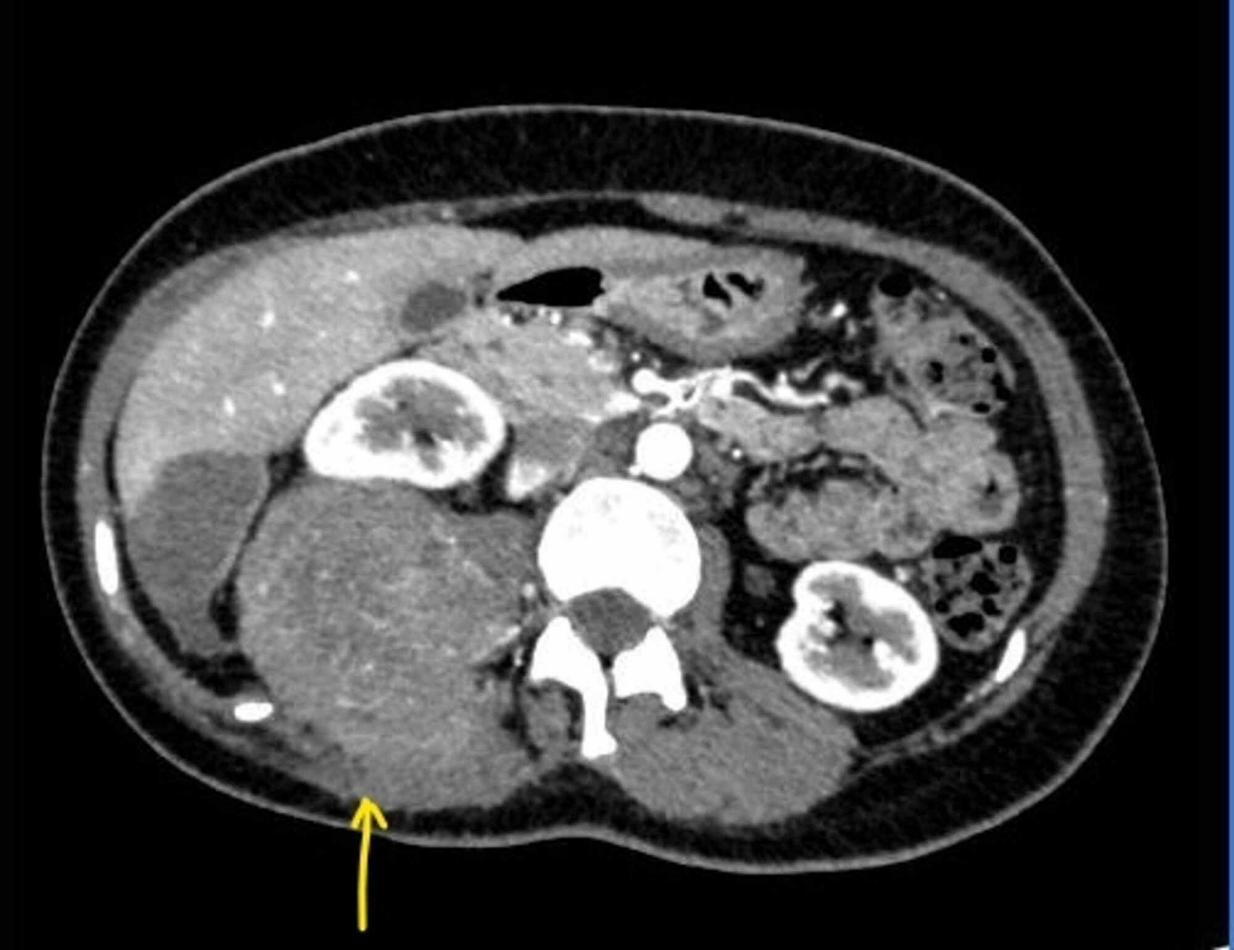
Contrast-enhanced CT image of tumor displacing right kidney.

**Figure 2:**
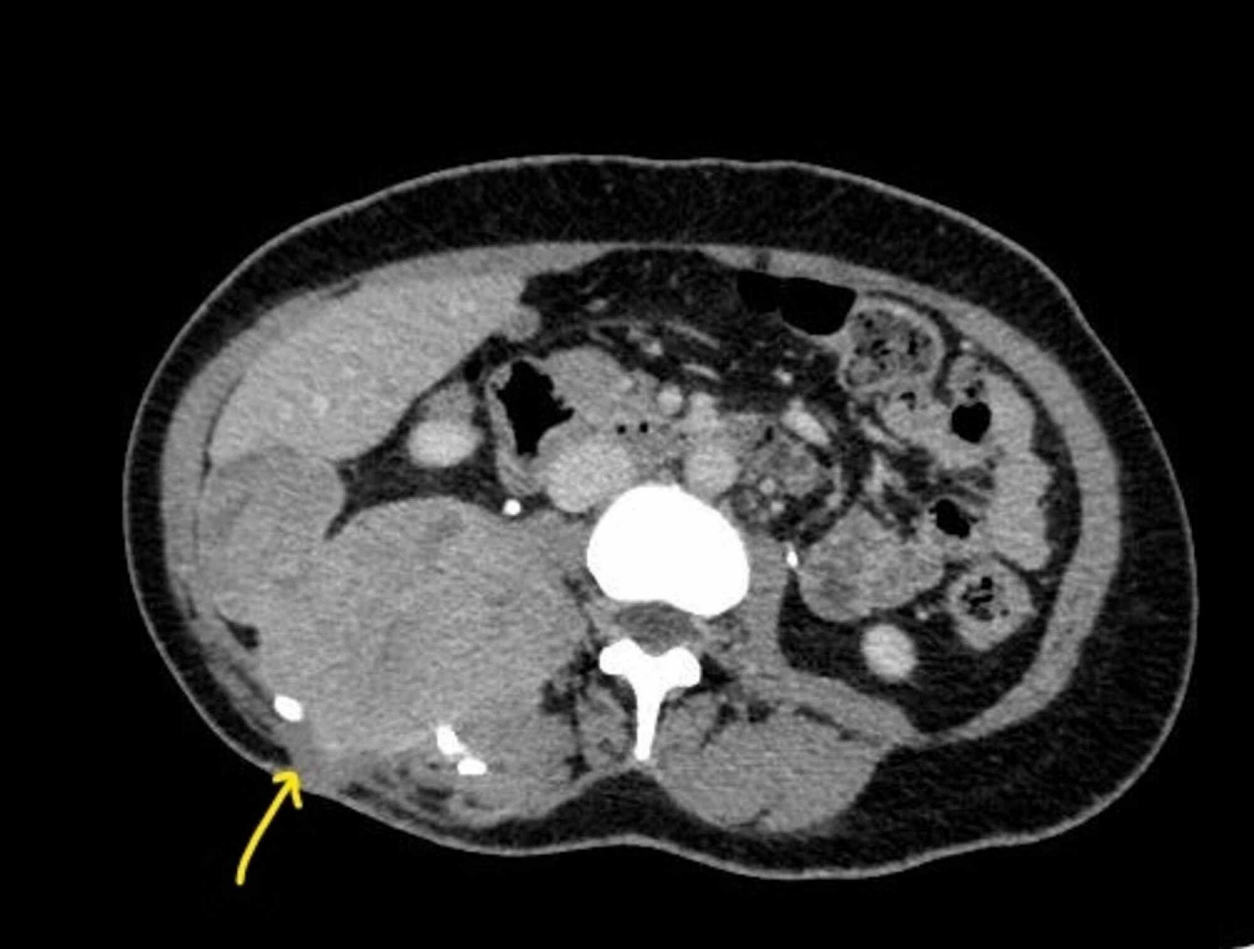
Contrast-enhanced CT image of tumor extending to posterior abdominal wall.

At the age of 27 years, she had another recurrence and presented with a large lump at the right lumbar region over the previous scar. There was a contrast-enhanced mass of 6.5 x 9.5 x 8.0 cm at the right loin in the contrast-enhanced CT scan (Figure 1). The lesion is situated at the subcutaneous tissue, a posterior abdominal wall that extended up to the lateral border of the right psoas muscle at the lower pole of the right kidney (Figure 2). The kidney could be identified separately, and the lesion appeared to abut the Gerota’s fascia. Prominent lumbar vessels were noted. The liver, spleen, and pancreas were normal with no ascites. It was consistent with the recurrence of the known fibrosarcoma.

 

A pathology report revealed a malignant spindle cell tumor with possibilities of either a leiomyosarcoma or fibrosarcoma FNCLCC (Federation Nationale des Centers de Lutte Contre Le Cancer) grade 3. It was infiltrating the psoas muscle, involving the deep margins. The tumor proliferation index is 30%. She completed neoadjuvant chemotherapy six cycles with IV doxorubicin 60 mg/m^2^ and IV cyclophosphamide 1,000 mg/m^2^ every three weeks. After six chemotherapy cycles, the tumor was radiologically stable, and clinically there was a very significant response. At the initial presentation in the second relapse, the patient’s WHO performance status was 3; at the end of four chemotherapy cycles, her WHO performance scale was 0. Definitive surgery was performed four weeks after the last chemotherapy. There were only necrotic tissues and were not clear well-demarcated margins. No active tumor cells were found. Since there were no solid tissues to be found, adjuvant radiotherapy was planned. Since the tumor size was large, we had a problem finding a spacer to locate at the tumor bed to keep the bowels away from the radiation field.

There was a six-month delay in supplying a spacer. Before radiotherapy, a follow-up assessment MRI scan showed a large heterogeneous irregular mass of 8.4 x 7.7 x 10.1 cm with hemorrhagic and fluid-filled areas within the mass, infiltrating the psoas muscle, extending up to the posterior abdominal wall. The right side erector spinae muscle, anterolaterally it abuts the liver with obliterated fat planes. No extensions to the spinal canal or extension into the pelvic bones, right kidney slightly rotated and shifted with slight obliteration of fat planes, no involvement of the right ureter, no hydronephrosis, and left kidney appeared normal with no evidence of any metastasis to the chest or liver and no significant lymphadenopathy. The tumor has recurred very early. The Multi-Disciplinary Team decision was no additional benefit of irradiating and decided to offer palliative care since there is no place for further chemotherapy due to severe toxicity and persistent side effects from previous chemotherapy.

However, the patient was given symptomatic relief with low dose metronomic chemotherapy with IV bevacizumab since it is a very vascular tumor. A Follow-up MRI scan showed that the mass was stable in size, 10.1 x 7.7 x 8.8 cm in completing chemotherapy. 

US abdomen and pelvis revealed a known malignant tumor of the abdomen's right side measuring 16.6 x 16.7 x 10.8 cm in size, mildly echogenic bilateral kidneys, and mild ascites. At this point staging, CT revealed pulmonary metastasis. The patient passed away while on metronomic palliative chemotherapy due to pulmonary embolism.

## Discussion

IFS is a tumor with a good prognosis, but primary surgery must be decided very carefully, and adequate tumor resection is essential to prevent a recurrence. These tumors tend to recur, and primary tumor control depends on the surgery and surgical skills. Albert J et al. [4], congenital IFS should be considered borderline tumors because its biological behavior is very close to surrounding normal structures. The tumor behavior is better than that of adult fibrosarcoma. Since the histologic diagnosis is not very conclusive in most cases, only long-term clinical follow-up confirms the benign or malignant nature of any individual tumor. This patient was diagnosed to have infantile fibrosarcoma and recurred after 12 years. There was no active follow-up in between, so we are not very certain whether it grew slowly over 12 years or the growth restarted after 12 years. The second recurrence was again after 15 years, and there was no proper follow-up over 15 years. Meantime she had a normal life with good performance. She was graduated and married and had a child. After the second recurrence, the nature of the tumor was transferred into a very aggressive malignant tumor. First recurred within a year but obtained good tumor control with anthracycline-based chemotherapy and alkylating agents. These tumors are very aggressive once transformed into the very aggressive type of tumor; They are rapidly growing with extreme pressure effects due to the tumor volume effect.

L Max Akmond et al. [5] explains in their meta-analysis that there is a minimal benefit with adjuvant chemotherapy and in contrast, we obtained good tumor regression following neoadjuvant chemotherapy; maybe the chemotherapy response to leiomyosarcoma at certain anatomical locations was not good such as Uterine Leiomyosarcoma as mention in the above study. We should not delay in further adjuvant radiotherapy; once the tumor starts behaving very aggressively. The treating clinician should understand the fact that active surveillance of primary tumor control is important. And also the tumor management should invariably be done with a multidisciplinary approach.

## Conclusions

This lady presented to us with a very aggressive Leiomyosarcoma at the right loin region, the second recurrence. Initially, it was mentioned as infantile fibrosarcoma diagnosed at birth and the first recurrence at the age of twelve years with histology remains unchanged. After 14 years, her tumor recurred very aggressively and progressed until she was treated with neoadjuvant chemotherapy and achieved a complete pathological response. There are important lessons we need to understand through this case report. Infantile Fibro Sarcomas should be treated with complete tumor resection with expert surgical skills to prevent a recurrence. Secondly, once a tumor recurs, the patient is benefited from neoadjuvant chemotherapy to achieve the best tumor control. Thirdly IFS can later transform into a very aggressive leiomyosarcoma where a multidisciplinary approach is essential with complete surgical excision with adequate tumor margins and adjuvant chemotherapy and radiotherapy.
